# Prediction of T stage in gastric carcinoma by enhanced CT and oral contrast-enhanced ultrasonography

**DOI:** 10.1186/s12957-015-0577-7

**Published:** 2015-05-19

**Authors:** Tao Yu, Xinling Wang, Zilong Zhao, Fan Liu, Xiaoting Liu, Yan Zhao, Yahong Luo

**Affiliations:** Department of Medical Image, Dalian Medical University Clinical Oncology College, No. 44 Xiaoheyan Road, Dadong District, Shenyang, 110042 China; Department of Medical Image, Liaoning Cancer Hospital and Institute, No. 44 Xiaoheyan Road, Dadong District, Shenyang, 110042 China; Department of Ophthalmology, The Fourth Affiliated Hospital of China Medical University, No. 4 Chongshan East Road, Huanggu District, Shenyang, 110032 China; Surgery Department, The First Affiliated Hospital of China Medical University, No. 155 Nanjing North Street, Heping District, Shenyang, 110001 China; Department of Gastrosurgery, Liaoning Cancer Hospital and Institute, No. 44 Xiaoheyan Road, Dadong District, Shenyang, 110042 China

**Keywords:** Gastric carcinoma, T stage, Enhanced CT, Oral contrast-enhanced ultrasonography

## Abstract

**Background:**

The aim of this study is to explore the values of enhanced CT and oral contrast-enhanced ultrasonography on preoperative T stage in gastric carcinoma.

**Methods:**

Forty patients with gastric carcinoma, including 27 males and 13 females, were confirmed by endoscopy, operation, and pathology. The median age of these patients was 49 years old (25 to 73 years). There were 19 cases of well-differentiated adenocarcinoma, 13 cases of poorly differentiated adenocarcinoma, 5 cases of signet ring cell carcinoma, and 4 cases of mucinous adenocarcinoma by pathology. All these patients were examined by both enhanced CT and ultrasound examination simultaneously 1 week before surgery. T staging in all these gastric carcinomas was carried out by enhanced CT or oral contrast-enhanced ultrasonography, respectively, or by both of them. The statistical difference between T stage by imaging and pathological T stage was analyzed.

**Results:**

In this study, there were 5 cases with T1 stage, 9 cases with T2 stage, 20 cases with T3 stage, and 6 cases with T4 stage by pathology; 5 cases with T1 stage, 7 cases with T2 stage, 22 cases with T3 stage, and 6 cases with T4 stage by enhanced CT imaging with an accuracy of 75.00%; 6 cases with T1 stage, 7 cases with T2 stage, 22 cases with T3 stage, and 5 cases with T4 stage by ultrasonography examination, with an accuracy of 77.50%; and 4 cases with T1 stage, 10 cases with T2 stage, 19 cases with T3 stage, and 7 cases with T4 stage by both enhanced CT imaging and ultrasonography examination, with an accuracy of 85.00%. The accuracy of T staging in gastric carcinoma by both enhanced CT and ultrasound was higher than that either by enhanced CT or by ultrasound, respectively (*P* < 0.05). The anastomosis degree of the gastric carcinoma between enhanced CT and ultrasonography was *κ* = 0.404.

**Conclusions:**

Combination diagnosis of enhanced CT and oral contrast-enhanced ultrasonography is helpful to improve the accuracy of T staging of gastric carcinoma before operations.

## Background

Gastric cancer is the fourth most common cancer in the world, and the mortality rate ranks second in both sexes worldwide. Although the incidence and mortality rates of gastric cancer have slowly declined in many countries, it is still a serious threat to the safety and health of patients [1]. Clinical studies have shown that the survival rates and the survival time of patients with gastric cancers are closely related to gastric wall invasion (T staging), lymph node metastasis (N staging), and distant organ metastasis of gastric tumors (M staging) [2]. If stomach cancer is accurately diagnosed at an early stage, followed by a timely surgery, there is a good chance for patients to be cured and to live for a long time. Even for patients with advanced gastric cancer at the later stages, obtaining an accurate diagnosis and precise staging, the survival time can be extended by chemotherapy or neoadjuvant chemotherapy. Therefore, the preoperative accurate evaluation for T staging of gastric cancer followed by clinical auxiliary therapy to gastric cancer patients relies on the precise imaging characteristics. It is essential to increase the survival rates and survival time of patients with gastric cancer. Undoubtedly, it is an important issue for clinical doctors [3,4].

Medical imaging has been the main method for staging of gastric cancer. Currently, the methods commonly used for gastric cancer staging are multidetector computed tomography (MDCT), MRI, endoscopic ultrasonography (EUS), abdominal ultrasound, PET-CT, and other methods [5]. Enhanced scanning of MDCT has become the preferred method for gastric cancer staging due to the high spatial resolution, tissue resolution, and three-dimensional reconstruction techniques. However, the shortcomings of MDCT in the staging of gastric cancer are limited values in the evaluation of T1 gastric cancer staging and gastric lymph node metastasis, with limited values of 44.4% to 83.7% and 64.0% to 78.2%, respectively [6,7]. Therefore, the combination of enhanced MDCT scanning and other imaging methods to enhance the accuracy of staging diagnosis of gastric cancer for T stage and N stage is undoubtedly an important strategy for gastric cancer therapy.

Enhanced MRI scanning has a higher scanning resolution on soft tissue than enhanced MDCT scanning; however, the longer scanning time is not conducive to overcome peristalsis of the stomach. Additionally, the relatively expensive fees also contribute to the restriction of MRI application on staging of gastric cancer. Recently, EUS has increasingly become the best method combined with stomach MDCT for T staging of gastric cancer because of its better resolution at the stomach level. However, due to limitations of ultrasound beam attenuation, T3 or T4 staging of gastric cancer is limited in EUS. Additionally, the discomfort caused by the examination also limited the application of EUS [8]. The ultrasound imaging method (transabdominal ultrasound) was the traditional technique for the examination of the stomach, but it was once discarded by clinical doctors because of the scan depth limitations of ultrasound imaging at that time and especially the reverberation effects and graphic distortions caused by the retained gas in water filling the stomach cavity. With the development of ultrasound equipment and an oral ultrasound contrast agent, oral echo-ultrasound contrast agents were used in clinics, which promoted the progress of ultrasound diagnostic techniques on gastric cancer. Meanwhile, it would provide a new method for T staging of stomach cancer by imaging [9,10].

In double contrast-enhanced ultrasonography (DCUS) applications, Zheng *et al.* thought there were no statistically significant differences (*P* > 0.05) between its accuracy of T staging (about 77.2%) and EUC’s accuracy (about 74.7%) [11]. Therefore, DCUS may be the best alternative method for EUS in combination with MDCT in the T staging of gastric cancer. In the clinical application of DCUS, Chen *et al.* thought, to ensure good premise stomach cavity filling, the accuracy rate of oral echo-type filling agent ultrasound imaging for gastric staging is about 78.6% while the accuracy rate of DCUS for gastric cancer staging is 86.7%, and there was no significant difference between them (*P* > 0.05) [12]. Thus, we believed that there is a similar accuracy for the oral echo-type filling agent ultrasound imaging for gastric cancer staging and DCUS, but DCUS is considered important in combination with enhanced MDCT scanning, then oral echo-type filling agent-enhanced ultrasonography and MDCT scanning. It is possible to have a higher gastric T staging of complementary imaging techniques in ‘combination’.

Thus, the study combined and applied two kinds of imaging methods, such as enhanced MDCT scanning and oral echo-type filling agent ultrasound imaging, to discuss the evaluation on the combined application of two kinds of imaging. The T stages of forty patients with gastric cancer were diagnosed before surgery, and the results were compared with the postoperative pathological T staging results. The value of applying the two kinds of methods to evaluate the preoperative T staging of gastric cancer was discussed to them.

## Methods

### General information

A total of 40 patients with gastric cancer in the Department of Gastric Surgery of Provincial Tumor Hospital of Liaoning Province were admitted and included between July 2012 and January 2013. All of the patients were confirmed by endoscopy examination and treated by surgery. The inclusion criteria were as follows: the patients were identified to have gastric cancer confirmed by gastroscopy examination, without serious heart and lung disease and iodine allergies, and they finally had an operation. Exclusion criteria were as follows: patients with severe cardiopulmonary disease and iodine allergies, and patients who cannot be operated in the future. The study has been approved by the Ethics Committee of Liaoning Cancer Hospital and Institute with trial registration number 2013055, and all patients signed a written informed consent. All patients were simultaneously given preoperative CT scan and ultrasound imaging examination 1 week before the operation. The interval of imaging examination and surgery was between 1 and 7 days, with a median time of 3.5 days.

### Scanning methods

#### Enhanced CT scanning

A GE Lightspeed 16 multi-slice spiral CT machine (GE Healthcare, Little Chalfont, UK) is used for enhanced CT scanning. Patients need to fast for 12 h before CT. In order to reduce gastrointestinal peristalsis, 15 mg of anisodamine was intramuscularly injected 10 to 20 min before the examination. In order to expand the stomach cavity, patients should take 2 to 3 g of gas-generating agent 10 min before the examination.

#### Ultrasound imaging

A GE Logiq 9 color Doppler ultrasonic diagnostic equipment (GE Healthcare, Little Chalfont, UK) is used for ultrasound imaging, and a convex array probe is selected with a frequency of 3 to 7 MHz. Patients should keep an empty stomach before ultrasound examination or keep fasting for 12 h before CT or 4 h after CT scanning. Five minutes before the examination, the patient is asked to drink about 500 to 800 mL of gastrointestinal ultrasonography echo-type ultrasound contrast agents (Dongya Corporation, Huzhou, Zhejiang). During scanning, if lesions are in the cardia, the supine position should be used; if lesions are in the gastric fundus, gastric body, or the gastric antrum, the right lateral position is used. When imaging is not satisfactory, the left lateral, half-sitting, sitting, and standing position could be used.

### Image observation

Ultrasonography diagnosis was carried out by two senior doctors who both participated in the ultrasound examination of the patient and assessed the T staging of gastric cancer. After the enhanced CT scanning was completed, another two senior associate professors in diagnostic radiology made a double-blind diagnosis of the pictures and evaluated T staging of gastric cancer.

### Pathological examination

After the surgery, the gastric specimens were fixed with formalin, embedded in paraffin, and cut into 4-μm-thick sections. All the sections were prepared for hematoxylin and eosin (HE) staining. More than one pathological expert observed the slices with an optical microscope and diagnosed the pathological T stage of gastric cancer according to TNM staging criteria by the American Joint Committee on Cancer (AJCC) in 2010.

### Statistical analysis

All the data used statistical software SPSS 10.0 (*χ*^2^ test). The sample size in the study was 40 cases, and the *χ*^2^ test was used to analyze the differences between them.

*P* < 0.05 meant there was a statistical significance. Goodness of fit was analyzed using consistency test. *κ* (consistent coefficient) ≥ 0.7 meant strong goodness of fit, 0.7 > *κ* ≥ 0.4 meant general goodness of fit, and *κ* < 0.4 meant weak goodness of fit.

## Results

### Clinical data

We studied a total of 40 patients in the Provincial Tumor Hospital of Liaoning Province, with a median age of 49 years (range 25~73 years). As seen in Table 1, there were 27 males and 13 females. There were 15 cases of gastric antrum cancer, 10 cases of whole gastric cancer, 6 cases of gastric body cancer, 5 cases of gastric antrum and gastric corpus carcinoma, and 4 cases of gastric fundus gastric cardia cancer. The pathological types were as follows: 19 cases of moderately differentiated adenocarcinoma, 13 cases of poorly differentiated adenocarcinoma, 4 cases of mucinous adenocarcinoma, and 5 cases of signet ring cell carcinoma. Borrmann types were as follows: type I, 6 cases; type II, 10 cases; type III, 14 cases; and type IV, 10 cases.

**Table 1:** **Clinical data of 40 cases of gastric cancer patients**

	**Clinical data**
Median age	49
Sex	
Male	27 (67.5%)
Female	13 (32.5%)
Tumor location	
Gastric antrum	15 (37.5%)
Whole stomach	10 (25.0%)
Gastric body	6 (15.0%)
Gastric antrum gastric body	5 (12.5%)
Gastric cardia and fundus	4 (10.0%)
Pathological types	
Moderately differentiated adenocarcinoma	19 (47.5%)
Poorly differentiated adenocarcinoma	12 (30.0%)
Mucinous adenocarcinoma	4 (10.0%)
Signet ring cell carcinoma	5 (12.5%)
Borrmann classification	
Type I	6 (15.0%)
Type II	10 (25.0%)
Type III	14 (35.0%)
Type IV	10 (25.0%)

### Comparison of T stage by imaging and pathology

**Figure 1:**
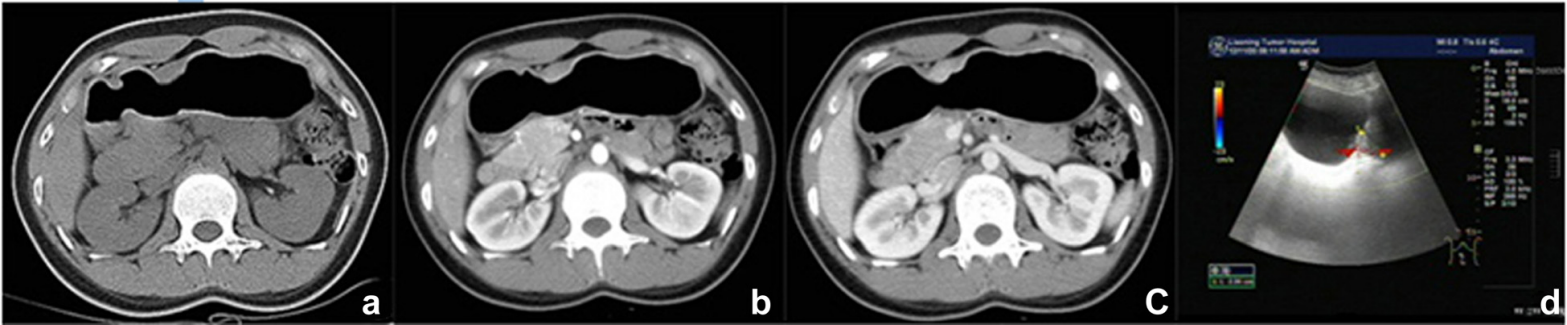
T1 tumors in lesser curvature of the gastric angle. **(a-c)** Enhanced CT scanning showed a clear gap surrounding the fat and smooth serosa. **(d)** Ultrasound imaging. Visible lesions were seen in the submucosa.

**Figure 2:**
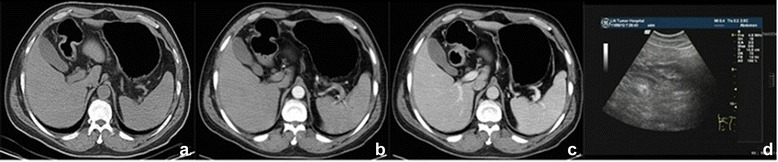
T2 gastric cancer in gastric antrum. **(a-c)** Enhanced CT scanning showed a clear gap surrounding the fat and smooth serosa. **(d)** The ultrasound imaging. Visible lesions invaded the muscle layer.

**Figure 3:**
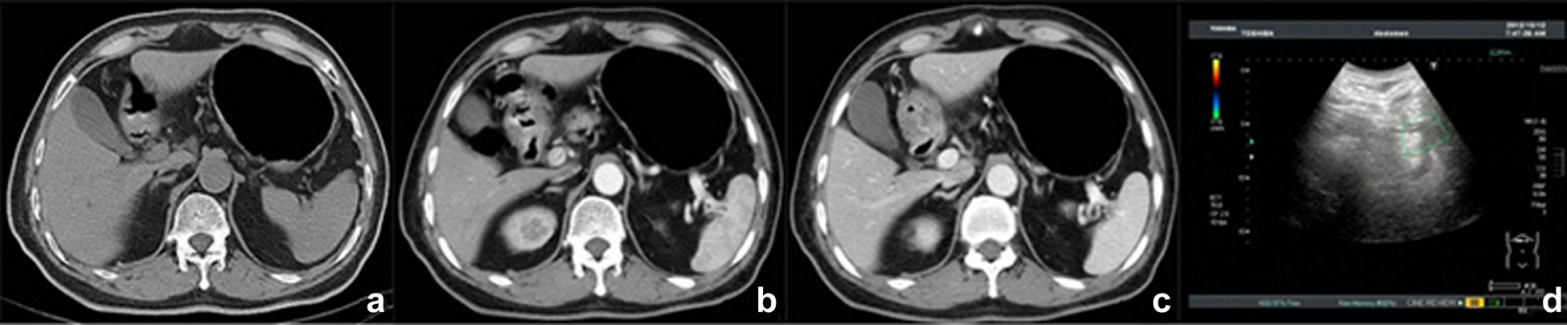
T3 gastric cancer in gastric antrum. **(a-c)** CT scanning. The nodules were visible around the fat space, and serosal surface was rough. **(d)** Ultrasound imaging. The visible lesions were invading outside the serosa.

**Figure 4:**
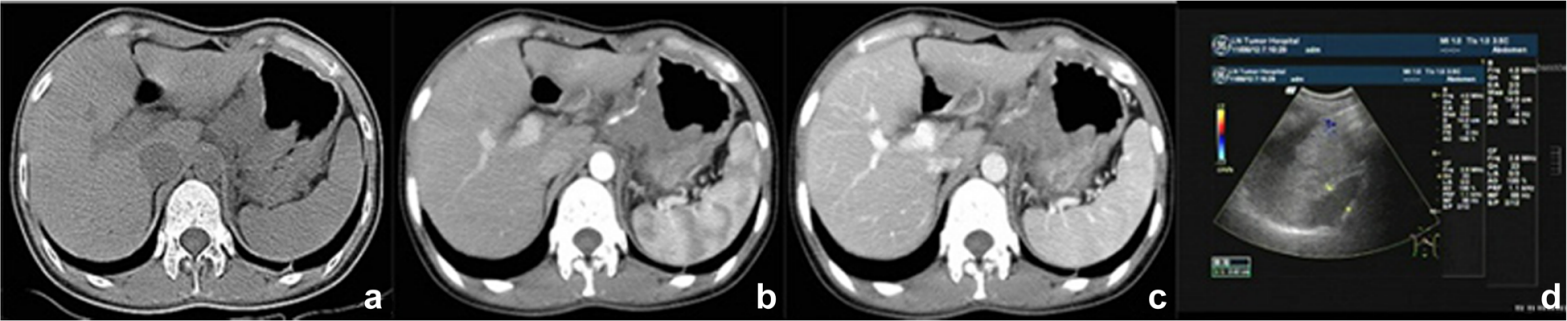
T4 gastric cancer in cardia and gastric fundus. **(a-c)** CT scanning. The gap around fat tissues disappeared, and the lesion invaded other structures such as the tail of the pancreas, left gastric artery. **(d)** Ultrasound imaging. It was visible that lesions invaded surrounding organs.

**Figure 5:**
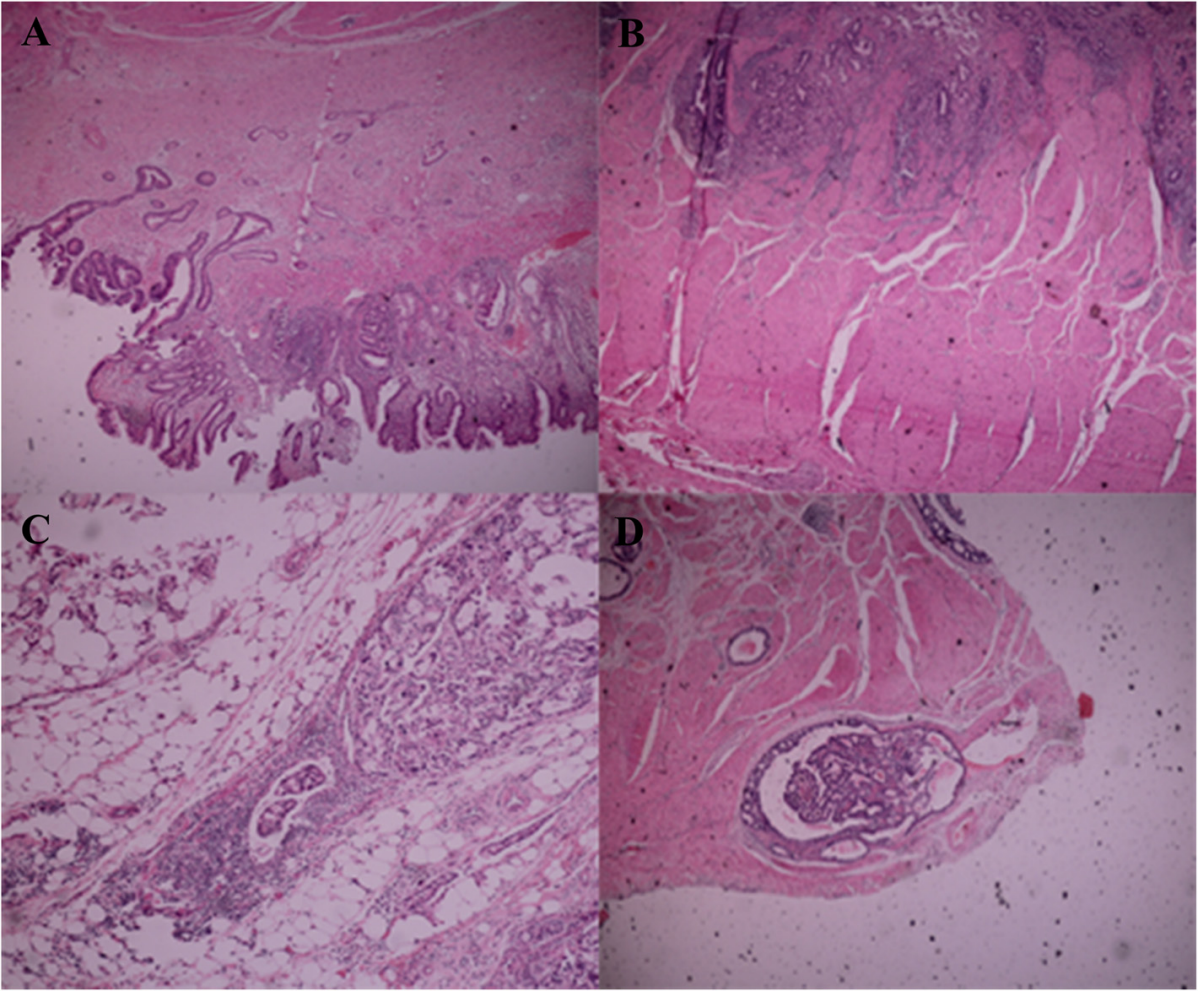
The pictures of pathological tissue slices. **(A)** Tumor invasion was limited to the submucosa. **(B)** Tumor invaded into inherent grassroots. **(C)** The lesions were invading outside the serosa. **(D)** Tumor invasion outside the serosa was invisible and accompanied by vascular thrombosis.

There were 40 cases of patients with gastric cancer in the group. The T stage was confirmed by pathological diagnosis after operation. Five cases were identified with clinical stage T1, 9 cases had tumor stage T2, 20 cases were in stage T3, and 6 cases were in stage T4. T stage was based on an ultrasound evaluation as follows: 6 cases of T1 stage, 7 cases of T2 stage, 22 cases of T3 stage, and 5 cases of T4 stage. A joint evaluation of T staging by CT and ultrasound analysis was as follows: 4 cases of T1 stage, 10 cases of T2 stage, 19 cases of T3 stage, and 7 cases of T4 stage (Figures 1, 2, 3, and 4). The comparison of T stage by pathological (Figure 5) and radiographic analysis in the group is shown in Table 2.

**Table 2:** **Comparison of 40 cases with gastric cancer by imaging and pathology**

**T stage by imaging**	**Pathological T stage**				**Total**
	**T1**	**T2**	**T3**	**T4**	
Enhanced CT scaning^*^					
T1	2	3			5
T2	3	4			7
T3		2	19	1	22
T4			1	5	6
Ultrasound imaging**					
T1	5	1			6
T2		5	2		7
T3		3	17	2	22
T4			1	4	5
Comprehensive imaging***					
T1	4				4
T2	1	7	2		10
T3		2	17		19
T4			1	6	7
Total	5	9	20	6	40

*P < 0.05; **P < 0.05; ***P < 0.05.

As shown in Table 2, the accuracy of T staging determined by CT, ultrasonography, and ultrasonography in combination with CT was 75.00%, 77.50%, and 85.00%, respectively. The enhanced CT scanning combined with ultrasound imaging had a higher accuracy in the evaluation of preoperative T staging of gastric cancer than the two methods used alone (*P* < 0.05).

According to Table 3, the Kappa consistency test result demonstrated that Kappa equaled 0.404, suggesting weak goodness of fit in the evaluation of T staging by enhanced CT scanning and ultrasound imaging of gastric cancer.

**Table 3:** **Comparison of both methods in the evaluation of gastric T stage in 40 cases with gastric cancer**

**Stages by ultrasound imaging**	**Stages by CT**				**Total**
	**T1**	**T2**	**T3**	**T4**	
T1	2	4			6
T2	3	3	1		7
T3			18	4	22
T4			3	2	5
Total	5	7	22	6	40

### Comparison of enhanced CT scanning and ultrasound imaging in T staging of gastric cancer

Forty cases of gastric cancer patients were included in the group. According to the results of enhanced CT scanning evaluation of T staging, 5 cases were in T1 stage, 7 cases in T2 stage, 22 cases in T3 stage, and 6 cases in T4 stage. T stage according to ultrasonography evaluation is as follows: 7 cases in T1 stage, 6 cases in T2 stage, 22 cases in T3 stage, and 5 cases in T4 stage. The comparison of both methods in the evaluation of gastric T stage is shown in Table 3.

## Discussion

Surgical operation is still the most effective way to cure gastric carcinoma. But for early gastric cancer, the tumor is limited to the mucosal layer, and endoscopic mucosal resection (EMR) can be implemented locally. Especially, in the last few years, the rapid development of endoscopic mucosal dissection (ESD) expands the indications for endoscopic surgery. For well-differentiated submucosally invasive carcinoma, tumors with a diameter of less than 3 cm, without lymph node metastasis, can be treated by endoscopic surgery. Because of the features such as less surgical trauma, fewer complications, and relatively simple operation, it is greatly promoted and widespread in clinical application. It is only performed to accurately assess the depth of tumor invasion and tumor size, which means the accurate T stage, and whether endoscopic therapy can be adopted in patients. Thus, preoperative T staging contributes to the appropriate surgical planning. The application of imaging methods for preoperative T staging of gastric cancer is important and with outstanding value for clinical treatment. In clinical diagnosis and treatment of gastric cancer, enhanced CT scanning is the most often applied imaging method for T staging of gastric cancer [6]. Recently, use of an oral echo-ultrasound contrast agent for ultrasound diagnosis of gastric cancer is considered as a very promising imaging method for T staging [13,14]. Thus, we discussed the value of the combined use of two methods (enhanced CT scanning and ultrasound imaging) in the evaluation of gastric cancer before surgery.

### CT staging of gastric cancer

Gastric spiral enhanced CT scanning can be used by two methods, filling the stomach with either water or air in the clinical setting. In this study, the inflator method is used. In the evaluation of T staging of gastric cancer, pathological T1 and T2 T staging of gastric cancer were evaluated by spiral enhanced CT scanning which could strengthen the difference between the gastric tumor and the stomach, but it had a limited evaluation capacity. In this study, for example, the results showed that the evaluation accuracy of pathological stages T1 and T2 for gastric cancer by spiral CT was about 42.86%, which was consistent with the reported data. In this group of patients, there were three cases of pathological T1 tumors diagnosed as T2 stage. In a retrospective analysis, two cases of endoscopic analysis showed that gastric cancer cells did not invade the muscles but more extensive infiltration of lymphocytes caused the local uplift in the stomach. There were three cases of pathological T2 stage gastric cancer which were diagnosed as T1 stage. Retrospective analysis results showed two cases of endoscopic gastric cancer cells only in ‘nests’ to change the infiltration to the muscle, and local uplift was not obvious in the stomach.

Spiral CT scanning has a greater advantage on the evaluation of T stage of gastric cancer in the pathological T3 and T4 stages, with an accuracy of about 89% to 98%. This is because the prominent feature of T3 gastric cancer on the spiral CT image is a blurred change in serosa and fat gap, and this imaging feature has a higher specificity. The T4 gastric prominent feature on the spiral CT is a violation of the surrounding organs, and this indicates that specificity is approaching 100% [15,16]. In this study, the results showed that the accuracy of spiral CT evaluation of pathological stages T3 and T4 for gastric cancer was about 92.31%, which was consistent with that reported in the literature. In this group of patients, there were two cases of gastric pathology in T2 stage which were mistakenly diagnosed as T3 stage. In a retrospective analysis, in one case, gastric cancer cells did not invade the serosa as evaluated by endoscopic examination, but obvious inflammation appeared outside the serosa. One case of pathological T3 stage was diagnosed as stage T4 gastric cancer (violations of the pancreas). A retrospective analysis showed the case was lean; fat tissue between the stomach and pancreas was meager. Although the endoscopic result showed that gastric cancer cells had not violated the pancreas, the CT scan showed that the gap around the fat tissue disappeared, which led to misdiagnosis [17].

### The staging of gastric cancer by ultrasonography

In the 1970s, oral contrast-enhanced ultrasonography is considered to be an important diagnostic imaging method for gastric cancer. Water, milk, and other agents were used to fill the stomach for clinical ultrasonography. But the time of filling the stomach cavity is short, the image contrast is low, and the repeatability is poor, all of which directly restrict the clinical application of ultrasound for the diagnosis of gastric cancer [18]. Then, EUS was used because it can clearly show the five-layer structure of the stomach from the mucosa, submucosa, muscle, serosa, and outer layer of serosa [19,20], and especially has a higher accuracy at the T1-T2 lesion installments [17]. The restrictive factors of EUS staging to gastric cancer are due to the attenuation of the ultrasonic beam which led to the lower accuracy of the T3-T4 staging of gastric cancer. Meanwhile, EUS examination was conducted on the basis of endoscopy, and the patient’s degree of acceptance of it was lower than that of the oral filling agent ultrasound [21].

In recent years, domestic new echo-type gastrointestinal agents appear as a significant aid in clinical application, which received acceptance of clinical and imaging doctors. Especially, agents are significantly simple, have effects on the dilatation of the stomach cavity, and have echo difference in gastric cancer, all of which make oral echo-type filling agent ultrasound imaging increasingly become an important method for T staging of gastric cancer [11]. Based on oral echo-type filling agent ultrasound imaging, whether using ultrasound imaging as an important supplement for MDCT T staging increasingly becomes a controversial issue [12,22].

In this study, oral echo-type filling agent ultrasound imaging T staging of gastric cancer was evaluated, and the overall accuracy reached 77.50%, which was consistent with that reported in the paper [12,23]. In this group of patients, five cases were in the pathological T1 stage of gastric cancer. The accuracy reached 100% by ultrasound staging. But for pathological T3 and T4 staging of gastric cancer, the accuracy of this study was about 80.76%, which is lower than that of the enhanced MDCT scanning (92.31%). We thought that the reason was due to the lesions being far from the ultrasound probe, resulting in a lower spatial resolution. On the other hand, it was because the movement of the stomach had interfered with the measurement of blood flow within the lesion [24,25]. While T3 to T4 gastric T staging is the advantage of enhanced MDCT scanning, therefore, we thought that oral contrast-enhanced ultrasonography and enhanced CT may have greater complementarity.

In this group of patients, two cases of gastric cancer in pathological T2 stage were diagnosed as T1 stage. The endoscopic examination showed that although gastric cancer cells only violated the submucosa, the nest distribution of gastric cancer cells forms a protrusion to the muscle, which caused the misjudgment of infiltration of the lesions by imaging. There were two cases of pathological T3 gastric cancer diagnosed as T2 stage. The endoscopic examination in one case showed that although gastric cancer cells penetrated the plasma membrane, the carcinoma outside the serosa showed a scattered distribution. There were two cases of pathological T4 stage of gastric cancer diagnosed as T3 stage. In one case, the lesions were located in the antral posterior wall which was away from the probe, and the lesions’ significant creeping infiltration, combined with the patient’s gastric motility, was very intense and had direct impact on the observation of the disease.

### T staging of gastric cancer by CT scan in combination with ultrasound imaging

Laparoscopic examination has a very important value for peritoneal metastasis in the abdominal cavity, which is the disadvantage for image examinations, such as CT or ultrasonography. Laparoscopy is applied for the staging of gastric carcinoma, which can effectively find peritoneal metastases before surgery, and can give an early open surgical resection. Moreover, it helps to accurately determine the T staging and contributes to the implementation of preoperative neoadjuvant chemotherapy. Therefore, the conventional CT imaging combined with laparoscopic examination is more helpful and meaningful for accurate preoperative staging [26].

Enhanced MDCT scanning is currently considered as the preferred method for T staging of gastric cancer in the world, but as a complementary staging, it remains to be further studied [21,27]. This study combined the findings of Zheng [11] and Chen *et al.* [12]. Enhanced MDCT scanning and oral echo agent ultrasound imaging were combined for the T staging of gastric cancer, and the accuracy reached 85.00%, which was consistent with that from Venkataraman [28]. The combination of enhanced CT scanning and ultrasound imaging has a higher accuracy in the evaluation of preoperative T staging of gastric cancer than any method used alone (*P* < 0.05).

The results in the present study demonstrated that T staging of gastric cancer based on CT scan and ultrasound imaging had a weak goodness of fit (Kappa consistency test *κ* = 0.404), which demonstrated that the overlapping of the evaluation by CT scan and ultrasound imaging to the results of T staging was not high. Thus, if one method made mistakes in the evaluation of T staging of gastric cancer, the other method could prompt existing errors that can improve the accuracy of T staging evaluation. For this group of patients, enhanced CT scanning had a higher accuracy (about 92.31%) in evaluating gastric cancer at pathological T3 and T4 stages. Ultrasound imaging had the accuracy of about 64.29% for pathological T1 and T2 stages of gastric cancer. Therefore, the combination of both methods is for complementary evaluation.

The limitations of this study are small sample sizes, especially in T1 stage, T2 stage, and T4 stage. We had used the appropriate statistical methods to eliminate bias; however, in order to get more accurate results, we need further studies to increase the sample size. Although there was a small number of patients listed in the study, there were 40 cases included. It was appropriate to use the *χ*^2^ test; thus, the conclusion was basically acceptable. Of course, the research is being done until now, and we will take larger sample sizes to make the results more reliable.

## Conclusions

In summary, the combination of enhanced CT scanning and oral contrast echo-enhanced ultrasonography imaging for preoperative T staging evaluation in gastric cancer patients is a simple clinical application therapy with a higher accuracy. It is an important and accurate method for gastric cancer therapy.
